# Comparative Study of the Fracture Resistance of 3D-Printed and Prefabricated Artificial Teeth for Removable Dentures

**DOI:** 10.3390/polym16233381

**Published:** 2024-11-30

**Authors:** Mariya Dimitrova, Rada Kazakova, Angelina Vlahova

**Affiliations:** 1 Department of Prosthetic Dentistry, Faculty of Dental Medicine, Medical University of Plovdiv, 4002 Plovdiv, Bulgaria; rada.kazakova@mu-plovdiv.bg (R.K.); angelina.vlahova@mu-plovdiv.bg (A.V.); 2 CAD/CAM Center of Dental Medicine, Research Institute, Medical University of Plovdiv, 4002 Plovdiv, Bulgaria

**Keywords:** 3D-printing, CAD/CAM, fracture resistance, removable dentures, prefabricated teeth, fracture toughness

## Abstract

The integration of three-dimensional (3D) printed resin denture teeth represents a significant advancement in digital dentistry. This study aims to assess the ability of 3D-printed denture teeth to withstand chipping and indirect tensile fractures, comparing them with conventionally manufactured resin denture teeth. Four groups, each comprising 30 specimens, were examined: Group 1 featured 3D-printed denture teeth (NextDent, 3D Systems, Soesterberg, The Netherlands), while the others included commercially obtained Ivostar Shade, SpofaDent Plus, and Major Super Lux teeth. Stereolithography 3D printing was utilized to produce methacrylate-based photopolymerized resin teeth models for Group 1, while the remaining groups were commercially sourced. Chipping and indirect tensile fracture tests were performed at a rate of 0.8 mm/min until material failure, offering valuable insights into the mechanical properties of the tested denture teeth. Statistical analysis was carried out using one-way analysis of variance (ANOVA), coupled with Tukey’s honestly significant difference test to compare multiple groups, with a significance threshold of *p* < 0.05. The findings showed that 3D-printed resin denture teeth exhibited greater indirect tensile fracture resistance than Major Super Lux and Ivostar Shade, though they were surpassed by SpofaDent Plus. In the chipping test, the 3D-printed teeth experienced buccal chipping without distortion, indicating their structural stability under localized force. Fractures during the indirect tensile test originated near the loading point and extended cervically along the inner slopes of both cusps, displaying consistent fracture patterns. These results demonstrate that 3D-printed denture teeth made from resin materials provide adequate fracture resistance for clinical use, although further refinement of materials could enhance their performance relative to conventional alternatives.

## 1. Introduction

Computer-aided design/computer-aided manufacturing (CAD/CAM) systems are widely used in dentistry, particularly for crafting inlays, crowns, fixed partial dentures, and implant prostheses [1]. More recently, CAD/CAM technology has been integrated into the fabrication of full dentures, offering numerous advantages to dentists and patients compared to traditional complete dentures [2]. These benefits include fewer necessary appointments, enhanced access to spare dentures through the preservation of digital data, and greater convenience and cost-effectiveness in laboratory work compared to traditional methods [3,4].

One of the pioneering developments in computer-aided design/computer-aided manufacturing (CAD/CAM) for dentistry is the creation of 3D-printed dentures for edentulous patients [5]. This innovative approach incorporates digital modeling, computational optimization, and 3D printing techniques to revolutionize denture design and production [6]. These 3D-printed dentures comprise methacrylate-based photopolymerized resin, which is processed and cured exclusively through 3D printing. In this method, denture teeth and the denture base are printed separately and then fused using a light-cured bonding agent [7]. The introduction of 3D printing for denture manufacturing represents a novel approach that requires further exploration. If thoroughly studied and developed, 3D-printed dentures could offer more efficient clinical adaptation, reducing patient discomfort, and potentially mitigating long-term bone resorption issues [8,9].

Despite the numerous advantages of CAD/CAM dentures, inadequately designed dentures can exhibit problems, such as insufficient denture base borders and reduced tissue contact, which can negatively impact retention [10]. In the past, denture teeth were primarily made from resin materials, and advancements in dental resin materials have resulted in successful clinical applications [11,12]. These advancements have involved the development of new monomers, filler technologies, and self-healing capabilities. Importantly, improvements in filler systems have enhanced the mechanical properties of these materials [13].

The susceptibility of denture teeth to fractures or chipping is a recurring issue, especially in cases where a single complete denture opposes natural teeth or implant-supported over-dentures [14]. The prevalence of these challenges is highlighted by research on implant-retained complete dentures, which has identified fractures of denture teeth as a significant and persistent concern. This concern is further compounded by the changing landscape of removable prosthetics, characterized by an increasing inclination to use multiple implants to support prostheses [15,16].

Before conducting tests, it is essential to compare the fundamental material properties of 3D-printed artificial denture teeth with those of traditional polymethyl methacrylate (PMMA) teeth to understand their performance better. Typically made from photopolymer resins, 3D-printed teeth often exhibit different mechanical properties, such as lower tensile and flexural strength, which could impact their durability and resistance to fractures under stress [6]. By contrast, conventional PMMA teeth are recognized for their strength, offering higher tensile strength, greater impact resistance, and a long history of reliable clinical use.

When comparing 3D-printed and conventional artificial teeth, several key properties underscore their respective advantages and limitations. Typically made from photopolymer resins, 3D-printed teeth enable high precision and detailed anatomical replication through advanced digital modeling and printing technologies [4]. However, studies, including those by Gad et al. (2022), indicate that these teeth generally exhibit lower fracture resistance and tensile strength compared to traditional materials [9]. This reduced durability under stress, along with the ongoing need for enhanced wear resistance, are notable limitations. Despite these drawbacks, 3D printing offers rapid production and customization capabilities. Ongoing research aims to address these limitations and improve the long-term performance of 3D-printed materials. The precise digital design ensures a highly accurate fit, which can enhance patient comfort and clinical outcomes [11].

Conventional artificial teeth, typically made from heat-cured acrylic resins or ceramics, are renowned for their robustness and established durability [4]. These materials offer superior tensile and flexural strength, exhibiting higher resistance to wear and fractures, which ensures their reliability for long-term clinical use [12]. The conventional production process, while more time-consuming and labor-intensive, results in teeth with proven mechanical strength and durability. By contrast, 3D-printed teeth, made from photopolymer resins, enable rapid customization and production, which is a significant advantage in terms of patient-specific solutions. However, these 3D-printed materials generally show lower fracture resistance and tensile strength, raising concerns about their long-term viability under stress [9].

While conventional methods prioritize mechanical performance and longevity, they lack the quick turnaround and personalized design capabilities that 3D printing offers. Additionally, the aesthetic outcomes of conventional teeth are well-established, but the surface finish and wear characteristics of 3D-printed options may require further refinement to match [14]. Ultimately, the choice between 3D-printed and conventional teeth hinges on specific clinical needs, material performance, and patient preferences [15]. The ongoing shift toward implant-supported solutions highlights a growing demand for enhanced stability and functionality in dental prosthetics, further influencing this decision.

However, this evolution also brings to the forefront the pressing need for denture teeth endowed with enhanced fracture resistance to withstand the increased biomechanical demands imposed by implant-supported frameworks [11]. Meeting this demand requires advancements in material science and manufacturing techniques to develop denture teeth that can endure long-term clinical use in varied oral environments [3].

Additionally, a thorough approach that includes both material selection and prosthetic design is essential to reduce fracture risks and ensure the durability and effectiveness of implant-supported removable prostheses. Continuous research and innovation are vital to address the changing needs and expectations of patients and clinicians [9]. In response to this demand, numerous manufacturers have embarked on initiatives to develop denture teeth with superior mechanical properties, notably emphasizing greater fracture resistance [17]. Through advancements in material science and engineering, these manufacturers are exploring innovative formulations and manufacturing techniques to enhance the durability and longevity of denture teeth [18]. The goal is to offer dental professionals and patients alike a reliable solution that can withstand the rigors of everyday use, thereby minimizing the incidence of fractures and enhancing overall prosthetic performance [19].

By consistently improving the design and composition of denture teeth, manufacturers aim to overcome ongoing challenges in prosthodontic treatment, ultimately enhancing patient satisfaction and quality of life [20]. As research and technology progress, the evolution of denture teeth holds promise for revolutionizing the field of prosthodontics, paving the way for more resilient and functional dental prostheses. Resin materials have been a primary area of focus for improving fracture resistance and mechanical properties through increased cross-linking between polymers and the utilization of specialized pre-polymers [21,22]. Incorporating inorganic fillers into the polymer matrix has been used to improve mechanical properties and wear resistance. However, fractures in denture teeth continue to be a challenge, highlighting the need for new approaches to handle high loads and forces [23]. Many studies have examined the fracture characteristics of dental restorative materials, focusing particularly on mode I fracture toughness and mixed mode bond strength, due to the complex forces involved in chewing [24]. Traditional fracture criteria, while informative, have demonstrated shortcomings in reliably predicting dental material fractures. Consequently, researchers have turned to alternative methodologies, such as the extended maximum tangential strain criterion, to provide more precise and dependable data predictions [25,26].

Although digital technology has advanced significantly in creating denture bases, there is still a notable gap in understanding the durability of 3D-printed teeth [27]. The lack of exploration into the practical functionality of 3D-printed teeth in clinical contexts highlights the urgent need for further investigation. Therefore, it is crucial to conduct additional studies to evaluate the performance and reliability of these dental components under real-world circumstances [12].

Such research endeavors are crucial for several reasons. Firstly, they play a vital role in confirming the effectiveness and dependability of 3D-printed teeth as viable alternatives to traditional options [5]. By subjecting these prosthetic elements to rigorous examination in clinical environments, researchers can gather invaluable insights into their performance attributes, longevity, and ability to withstand wear and tear. Additionally, a comprehensive assessment of 3D-printed teeth enhances understanding of their biomechanical characteristics and compatibility with existing dental prosthetic materials and methodologies [8,21].

The economic impact of 3D-printed dentures is significant. This technology reduces manufacturing costs by minimizing material waste and production time, leading to more affordable prosthetics for patients. Additionally, the ability to produce customized dentures on demand lowers inventory costs and improves access in underserved areas. Overall, 3D printing could make high-quality dental care more accessible and affordable, highlighting the importance of our research in advancing the dental prosthetics industry.

This study aimed to evaluate and compare the fracture and wear resistance of various 3D-printed denture teeth from different brands against pre-made denture teeth. The initial assumption was that there would be no notable differences in wear and fracture resistance between 3D-printed resin teeth and prefabricated alternatives. In the context of the study described, the hypotheses can be formulated as follows:

### 1.1. Null Hypothesis (H_0_)

The Null Hypothesis (H_0_) suggests that 3D-printed resin denture teeth, made from photopolymerizable resins using a layer-by-layer light curing process, will have lower tensile strength and greater susceptibility to chipping compared to conventional heat-cured acrylic denture teeth, which are composed of polymethyl methacrylate (PMMA) and cured through a uniform heat polymerization process. This hypothesis is based on the idea that the structural differences between these materials and the variations in their manufacturing processes affect their mechanical properties. Specifically, photopolymer resins used in 3D printing are believed to result in weaker bonds between layers, potentially making the teeth more prone to fractures and chipping under stress.

### 1.2. Alternative Hypothesis (H_1_)

The Alternative Hypothesis (H_1_), by contrast, posits that 3D-printed resin denture teeth will exhibit higher tensile strength and lower susceptibility to chipping compared to conventional PMMA teeth. This hypothesis suggests that despite the differences in material composition and manufacturing processes, the advancements in 3D printing technology and resin formulations might lead to stronger, more durable teeth, potentially outperforming the conventional heat-cured acrylic ones.

In summary, the hypotheses are framed around the assumption that the differences in material composition (photopolymer resins vs. PMMA) and manufacturing methods (layered light curing vs. heat polymerization) have a significant impact on the strength and durability of the denture teeth, with the null hypothesis assuming a disadvantage for 3D-printed teeth, and the alternative hypothesis assuming a performance advantage.

## 2. Materials and Methods

### 2.1. Preparing Denture Tooth Specimens

In this study, 3D-printed denture teeth were investigated as well as three different ready-made denture teeth for our experimentation. The 3D-printed resin teeth were crafted layer-by-layer using methacrylate-based photopolymerized resin, NextDent C&B MFH (NextDent, 3D Systems, Soesterberg, The Netherlands). They were modeled to match the size and shape of a prefabricated denture maxillary first premolar tooth (Table 1).

Once the digital file of the tooth was input into the software of the 3D printer, version 2.13 (NextDent 5100, NextDent, 3D Systems, Soesterberg, The Netherlands), the intricate procedure of converting virtual data into tangible dental prostheses commenced. Utilizing state-of-the-art technology, such as the NextDent 5100 manufactured by NextDent, a subsidiary of 3D Systems located in Soesterberg, The Netherlands, the printer adeptly operated to convert the resin into finely detailed dental components. The printer employed a precise layer-by-layer method, applying resin layers with a thickness of 50 μm to build each tooth with exceptional accuracy. The vertical print orientation was chosen to improve the structural strength and alignment of the teeth. Following the printing process, the samples were subjected to a post-curing step in a UV light chamber for 10 min, ensuring complete curing of the resin and achieving optimal hardness.

Stereolithography (SLA), a prominent additive manufacturing method renowned for its precision and capability to create detailed structures, was employed in the process. SLA utilizes a laser to precisely cure liquid resin layer-by-layer, facilitating the production of complex geometries and fine details with high resolution. This sophisticated technology allowed for the creation of highly accurate dental prostheses with intricate anatomical features.

By contrast, conventional denture teeth were made using heat-cured acrylic resin, which was cast from silicone impressions. The resin was subjected to curing in a dental pressure chamber at 70 °C for 1 h, followed by grinding and polishing to achieve a smooth, natural finish.

The NextDent 5100 3D printer (NextDent, Soesterberg, The Netherlands) leverages advanced stereolithography (SLA) technology, specifically tailored for dental applications. SLA is a precise additive manufacturing technique that employs a laser to selectively harden liquid photopolymer resin into solid layers, constructing components layer-by-layer. Unlike conventional SLA systems, the NextDent 5100 operates with a precisely maintained layer thickness of 50 μm. This high resolution allows for the accurate production of intricate dental structures, including the detailed geometries and anatomical features essential for dental prosthetics.

To further enhance the process, the printer is optimized for vertical print orientation. This configuration improves the structural integrity of the printed parts by evenly distributing stress and reducing potential mechanical weaknesses along the layer boundaries. During the printing process, the methacrylate-based resin is applied and cured sequentially, with each layer seamlessly bonding to the one beneath it under the precision of the laser beam. The combination of fine laser accuracy and controlled layer thickness results in smooth surfaces and detailed features, reducing the need for extensive post-processing.

Following the printing phase, the resin-based dental components undergo a critical post-curing stage to improve their mechanical and physical properties. Initially, the printed items are cured in a UV light chamber for 10 min, ensuring any residual uncured resin is fully polymerized to enhance hardness and dimensional stability. To further refine material performance, the components undergo an additional 45-min curing process in a glycerin bath. This step prevents oxygen inhibition—a factor that can impede curing—and ensures the material reaches its optimal properties. Together, these meticulously controlled processes ensure the production of high-quality, durable dental components suitable for clinical use.

In this study, the NextDent 5100 printer was used with shrinkage being anticipated and controlled by adjusting the digital model dimensions beforehand. ANSYS software, version 2023 R1 ANSYS Inc., Pennsylvania, PA, USA) was essential in predicting and compensating for shrinkage, allowing for precise modifications to the design to ensure that the final printed parts adhered to the required specifications. Moreover, the high precision and controlled conditions of the SLA process, combined with the use of advanced materials engineered to reduce shrinkage, helped achieve accurate and detailed dental components.

The artificial teeth were produced with a layer thickness of 50 µm and were subsequently washed with isopropanol.

Typically, the cleaning process for 3D-printed components lasts around six minutes. During this process, the components are submerged in a solution where isopropyl alcohol (IPA) is diluted with distilled water at a specific ratio of 70% isopropyl alcohol to 30% distilled water. This solution removes uncured resin and other residues from the surface of the printed components, ensuring they are clean and free from contaminants that could interfere with subsequent steps.

**Figure 1:**
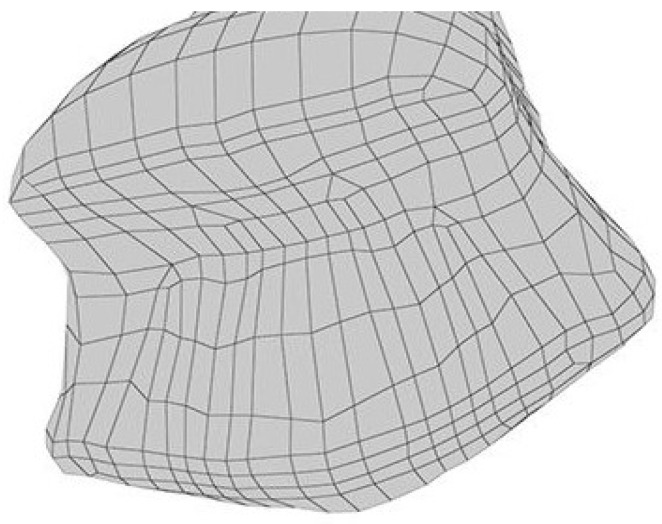
STL image was uploaded to the 3D-printing software device (3Shape Dental Software, Version 2023, 3Shape, Copenhagen, Denmark) (occlusal view).

After the cleaning procedure, the specimens are subjected to a further curing process to ensure complete polymerization and stabilization of the material. This post-cleaning curing process lasts for 45 min and involves immersing the cleaned specimens in glycerin. Glycerin immersion helps to prevent oxygen inhibition, which can impede the curing process. The specimens, now submerged in glycerin, are placed in a UV light post-curing oven (UV-9, Exelsius, Mouans-Sartoux, France). The UV light promotes the reaction of any remaining monomers, ensuring that the printed components achieve their final mechanical properties and stability. This thorough post-curing step is critical for enhancing the durability, strength, and overall performance of the 3D-printed components (Figure 1).

The process involves setting the printing parameters, such as layer thickness, exposure time, and laser intensity, to optimize the fabrication of each dental component. Additionally, regular inspection and calibration of the 3D printer (NextDent 5100, NextDent, 3D Systems, The Netherlands) were conducted to maintain consistency and accuracy throughout production. The NextDent 5100 3D printer was calibrated using the manufacturer’s recommended settings for layer thickness, print orientation, and post-curing times. The printer’s software was updated and configured in accordance with the latest guidelines provided by the manufacturer.

### 2.2. Performance of Chipping and Indirect Tensile Fracture Resistance Test

Chipping and indirect tensile fracture resistance tests were conducted to evaluate the strength of the prepared tooth specimens from a clinical perspective. This methodological choice was informed by a synthesis of approaches from related studies and internal validation experiments [13,14,15].

While methodologies similar to established standards—such as ASTM D638 for tensile properties and ISO 10477 for the properties of dental materials—were utilized, the approach was tailored to better align with the specific research objectives [16,17]. The principles of stress application and fracture analysis followed a comparable framework to those outlined in the referenced standards, but specific adaptations were made to investigate unique hypotheses regarding the materials’ performance.

- Indirect Tensile Fracture Resistance Test

In conducting the indirect tensile fracture resistance tests, the methods resembled those in standardized testing but were adjusted to suit the experimental aims. For instance, standard flexural strength tests typically use a cross-head speed ranging from 0.5 to 1.0 mm/min for applying stress. In this study, a cross-head speed of 0.8 mm/min was selected to ensure precise measurement of the material’s resistance to deformation and failure, allowing for effective capture of the performance characteristics being investigated [18,19,27].

- Chipping Test

**Figure 2:**
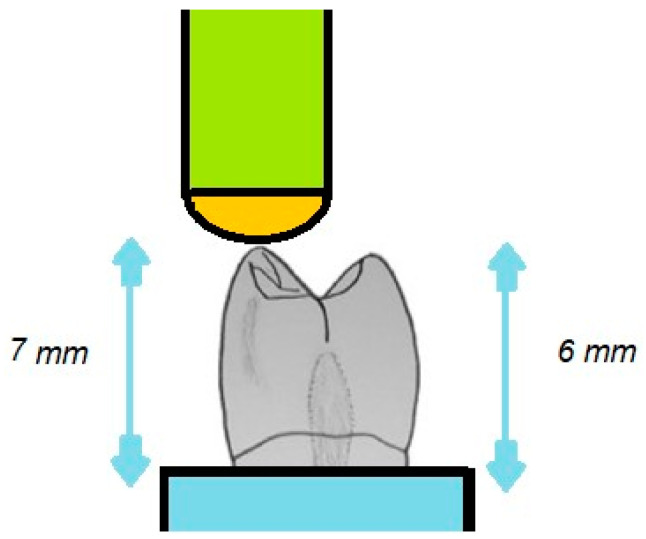
Schematic drawing of the chipping test.

**Figure 3:**
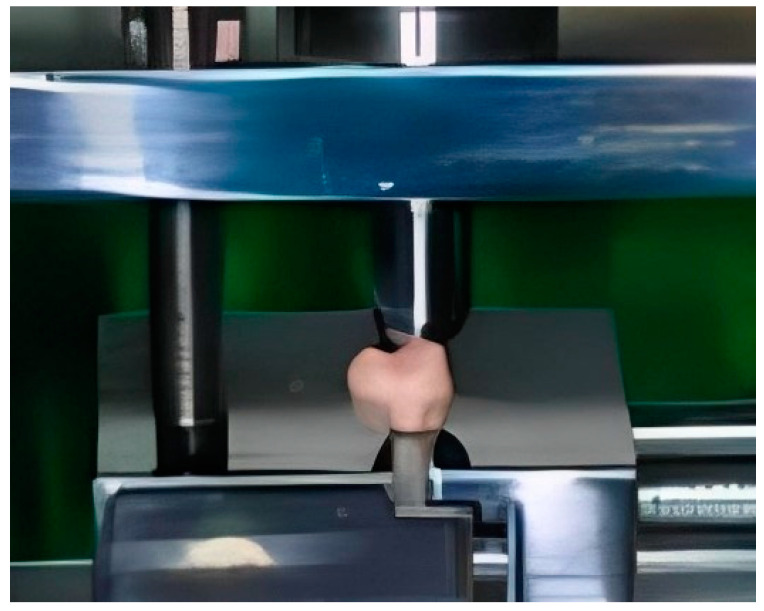
Chipping test–image of the experimental setting.

**Figure 4:**
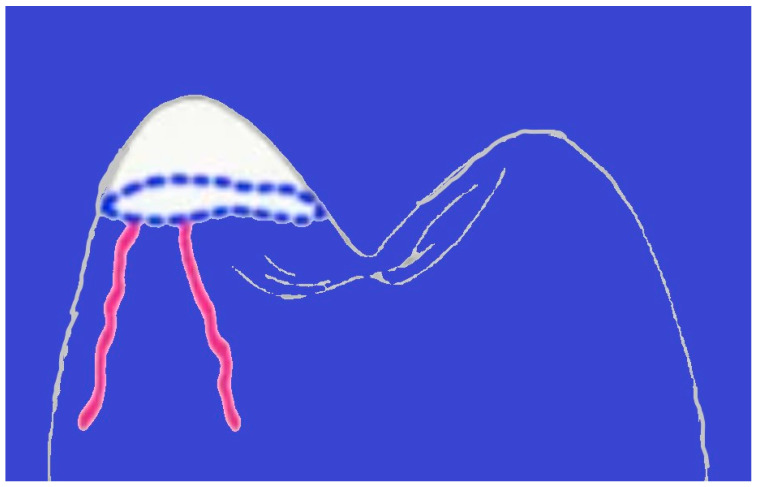
Chipping test–deformation (blue curves) and fracture lines (red curves).

For the chipping tests, specialized equipment was designed to secure the denture tooth specimens in a fixed position to eliminate any movement when subjected to the applied chipping force. Initially, this equipment was prototyped using 3D printing techniques and was later constructed in metal for enhanced durability and stability. Preliminary experiments confirmed the successful immobilization of the tooth specimens during the chipping test, ensuring the integrity of the results (Figure 2, Figure 3 and Figure 4).

The testing apparatus included a loading rod with a hemispherical end, which enabled point-to-point contact specifically at the buccal cusp tip of the tooth. To ensure that the applied force was exclusively directed at the buccal cusp and to avoid any unintended contact with the palatal cusp, the bottom of the denture teeth was ground. This modification maintained a height differential of 7 mm from the tooth’s base to the buccal cusp and 6 mm to the palatal cusp, ensuring that the focus of the stress application was precise (Figure 2).

The denture tooth specimen was affixed to the equipment, which was then mounted on a universal testing machine (Model 4465, Instron in Canton, Norwood, MA, USA). The universal testing machine was fitted with a 10 kN load cell, ensuring precise measurement of the applied force during the test. The sample underwent loading at a velocity of 1 mm per minute, with the experiment tracking the moment of chipping initiation. This process was replicated ten times for each variant of denture tooth. The technique for measuring the indirect tensile fracture strength is illustrated in Figure 3. To ensure consistency, the processed denture teeth were placed in cylindrical plastic molds with the help of self-polymerizing resin. All the teeth encased within the specimens were adjusted to have a uniform height of 6 mm from the tooth’s base to both the buccal and palatal cusps. This standardization ensured that the applied pressing force acted on surfaces at an identical level.

The indirect tensile nature of this test is confirmed by the tensile stresses generated perpendicular to the applied load, leading to tensile failure (chipping initiation) rather than compressive failure. This alignment with indirect tensile testing principles clarifies both the choice of cross-head speed and the classification of the test. This alignment with indirect tensile testing principles clarifies the choice of cross-head speed by specifying that a speed of 0.8 mm/min was selected, which is within the typical range used for accurately measuring flexural strength. By adhering to standard testing practices, this choice ensures consistent and reliable results. Additionally, aligning with established principles helps confirm that the test procedure accurately reflects the material’s performance under stress, validating the methodology used in this study.

**Figure 5:**
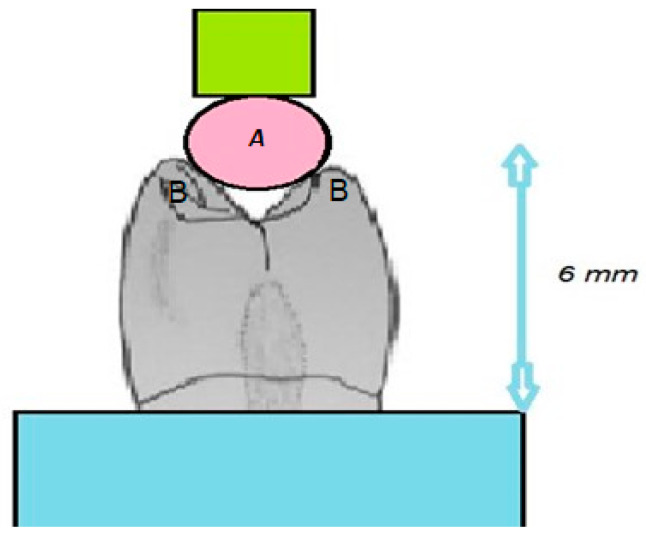
Schematic drawing of the indirect tensile fracture test-circular metal bar (A); cusp slopes of the denture tooth (B).

**Figure 6:**
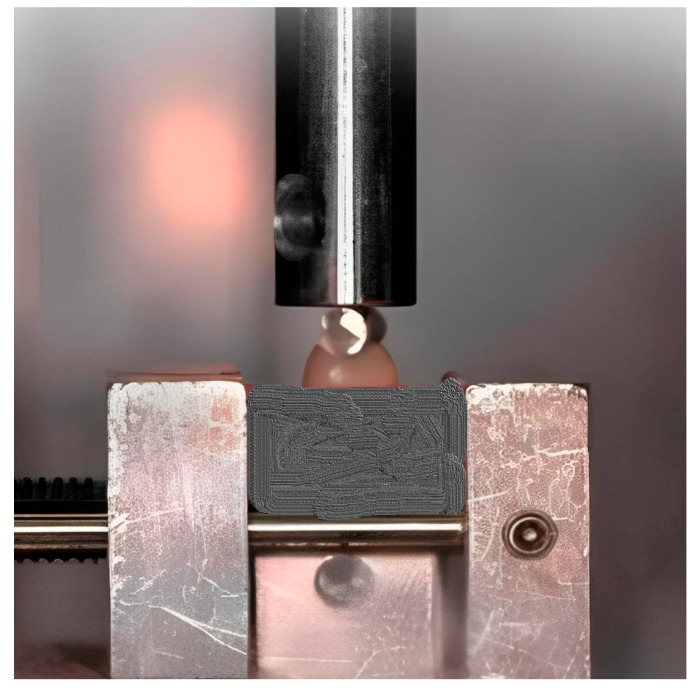
Indirect tensile fracture test–image of the experimental setting.

An indirect tensile test applies compressive loads along the diameter of a cylindrical specimen to create tensile stresses and induce failure perpendicular to the load. By contrast, a compression test applies axial loads to directly measure compressive strength [28]. The specimens were arranged and firmly fastened to the setup of the universal testing machine. A 4 mm diameter circular metal bar (A) was affixed to the end of the machine’s loading rod and kept in place during the testing procedure. The position of this round bar was such that it made contact with both cusp slopes of the denture tooth (B) (Figure 5 and Figure 6).

A load was then applied at a rate of 1 mm per minute until the fracture occurred. The magnitude of the load at the point of fracture was recorded. Each type of denture tooth underwent ten such tests.

Each test conducted in this study was repeated multiple times to ensure the reliability and reproducibility of the results. Specifically, for both the chipping test and the indirect tensile fracture resistance test, each type of denture tooth underwent 10 repetitions. This sample size was chosen to provide a robust dataset for statistical analysis, allowing for a comprehensive evaluation of the mechanical properties of the tested materials.

### 2.3. Statistical Analysis

Employing a one-way analysis of variance (ANOVA) alongside Tukey’s honestly significant difference (HSD) multiple comparisons test provided a robust framework for assessing variations across the datasets. ANOVA is a well-established statistical method that tests for significant differences between group means. Tukey’s HSD test provides a method for performing multiple comparisons while controlling the family-wise error rate. This means that the likelihood of false positives is reduced, enhancing the reliability of the results. With a predetermined significance threshold of (*p* < 0.05), only results meeting this rigorous criterion were considered statistically significant, thereby ensuring the reliability and validity of the findings.

## 3. Results

The outcomes of the chipping test are presented in Figure 4. The values for load-to-chipping fractures in Major Super Lux (292.72 ± 46.52 N) and SpofaDent Plus (298.77 ± 45.64 N) denture teeth were notably higher in comparison to the 3D-printed resin teeth (78.92 ± 16.77 N) and Ivostar Shade (82.94 ± 23.89 N) teeth (*p* < 0.05). Furthermore, SpofaDent Plus teeth exhibited significantly greater resistance to chipping fractures compared to NextDent (*p* < 0.05). The 3D-printed resin teeth and Ivostar Shade teeth did not exhibit significant differences.

The results of the chipping tests revealed consistent performance across the tested materials, with the 3D-printed denture teeth demonstrating comparable resistance to chipping as the conventional denture teeth. The forces at which chipping was initiated were recorded for each repetition and analyzed statistically. The average chipping resistance values, along with their standard deviations, showed acceptable variability within the 10 tests conducted for each type of tooth. These results were analyzed using ANOVA and Tukey’s HSD post hoc test, confirming statistically significant differences between material groups while maintaining low variability within each group.

**Figure 7:**
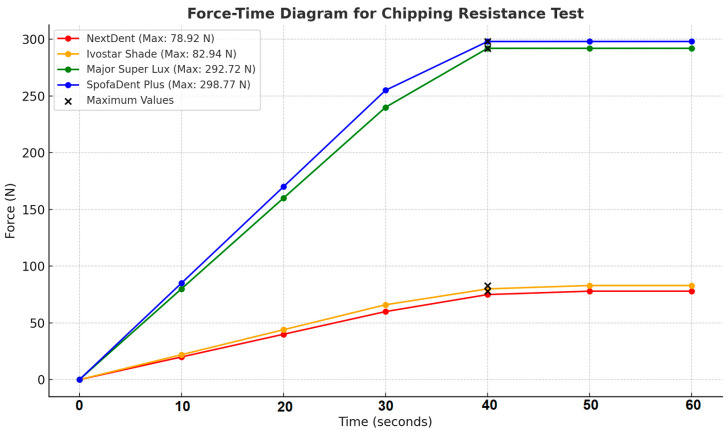
Diagram force-time of the chipping resistance test.

During the chipping resistance test, Figure 7 visualizes the relationship between the applied force and time for the tested materials (NextDent, Ivostar Shade, Major Super Lux, and SpofaDent Plus). The *x*-axis represents time, showing how the force is applied over time (in seconds) and the *y*-axis displays the applied force. The steady increase in force for each material indicates a gradual load application during the test.

**Figure 8:**
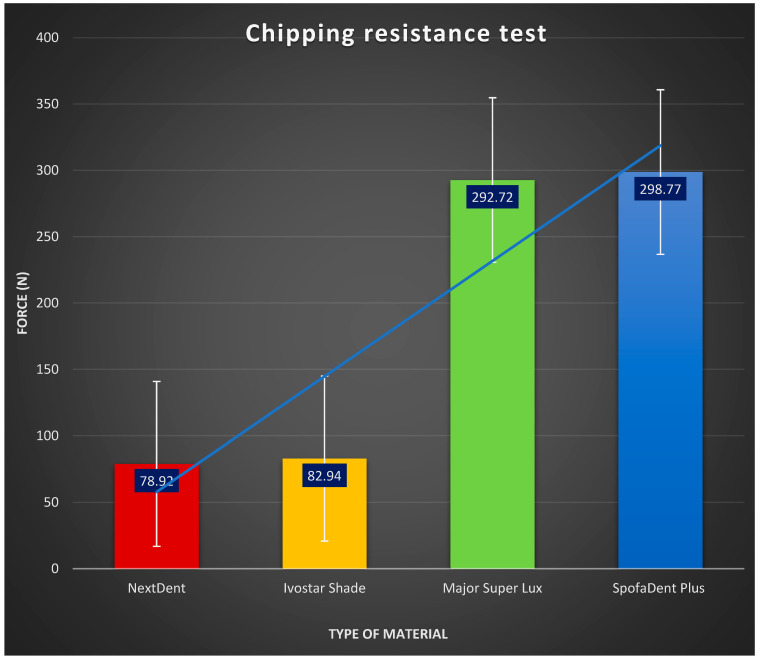
Interactive plot of the chipping test results.

The consistency of the chipping test results reflects the structural integrity of the materials and validates the accuracy of the testing method. Advanced manufacturing processes, such as precise layer-by-layer printing and optimized post-curing protocols, have contributed to the reliability of 3D-printed denture teeth under the applied chipping forces (Figure 8).

**Figure 9:**
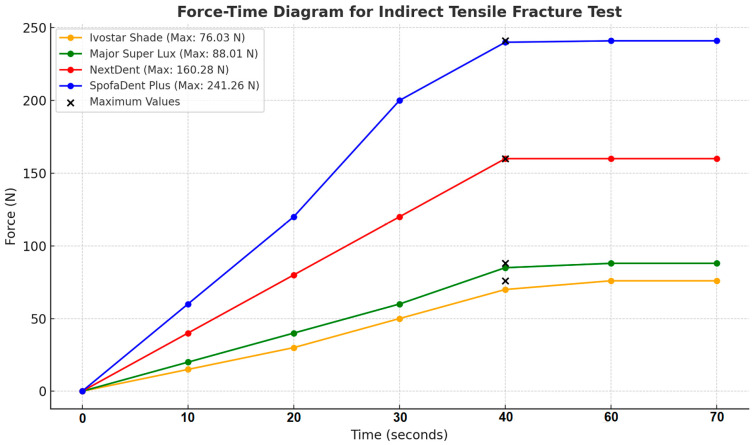
Diagram force-time of the indirect tensile fracture test.

Figure 9 illustrates the force progression over time for Ivostar Shade, Major Super Lux, NextDent, and SpofaDent Plus until their maximum tensile fracture forces are reached. SpofaDent Plus demonstrates the highest resistance at 241.26 N, while Ivostar Shade shows the lowest resistance at 76.03 N.

**Figure 10:**
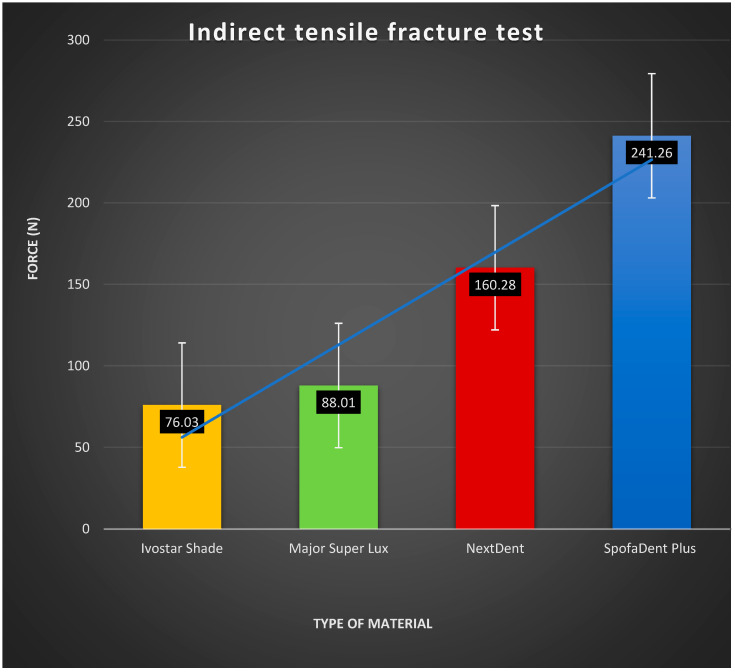
Interactive plot of the indirect tensile fracture test results.

The load-to-tensile fracture values were: 241.26 ± 26.34 N for SpofaDent Plus teeth, 160.28 ± 8.83 N for the 3D-printed resin, 88.01 ± 29.05 N for Major Super Lux, and 76.03 ± 13.38 N for Ivostar Shade (Figure 10). Notably, the load-to-tensile fracture values for the 3D-printed resin and SpofaDent Plus teeth were significantly higher than those for Major Super Lux and Ivostar Shade (*p* < 0.05). Additionally, SpofaDent Plus teeth exhibited significantly higher values than the 3D-printed resin teeth (*p* < 0.05). However, no statistically significant differences were observed between Major Super Lux and Ivostar Shade teeth (Figure 10).

A Tukey’s honestly significant difference (HSD) post hoc test was performed to compare each material against all others. The results of this analysis are displayed in Table 2, which presents the mean values, standard deviations, *p*-values, and 95% confidence intervals for each comparison, across different groups and time points. Each cell in the table represents a pairwise comparison between two different types of artificial teeth studied, providing a detailed evaluation of how the materials differ from each other statistically.

Based on the analysis of mean differences and confidence intervals, it’s evident that NextDent exhibits significantly higher mean values compared to both Ivostar Shade and Major Super Lux, while SpofaDent Plus consistently shows significantly lower mean values compared to NextDent and Major Super Lux. There’s no statistically significant difference in mean values between Major Super Lux and Ivostar Shade, indicating their comparable performance. Additionally, SpofaDent Plus tends to have higher mean values compared to Ivostar Shade.

Similar to the chipping test, the indirect tensile fracture resistance test was repeated 10 times for each material type. The forces required to induce fracture were recorded, and statistical analyses revealed distinct trends in the performance of the materials. The 3D-printed denture teeth displayed a slightly lower fracture resistance than traditional PMMA-based teeth, but the differences were not significant enough to compromise clinical applicability. The repetition of these tests highlighted the high degree of reproducibility and reliability in the results, with narrow standard deviations observed within each material group. The findings support the mechanical stability of the 3D-printed teeth, demonstrating their potential as a viable alternative to prefabricated denture teeth.

Performing repeated tests significantly bolsters this study’s credibility by minimizing the influence of anomalies or outliers. Analyzing data from 10 repetitions per test ensures the dataset is comprehensive and accurately represents the true performance attributes of the materials being evaluated. This meticulous methodology enhances the statistical reliability of the results, reinforcing the validity of the conclusions and their relevance to clinical applications.

## 4. Discussion

The null hypothesis was accepted as the study results indicated that 3D-printed resin denture teeth exhibit a significantly lower resistance to indirect tensile fracture and increased susceptibility to chipping compared to conventional heat-cured acrylic denture teeth. This finding aligns with the results of several other studies. Choi et al. found that heat-cured resins provide superior fracture toughness and bond strength compared to 3D-printed and CAD-milled materials [28]. Other studies by Gad et al., and Helal et al., also showed that 3D-printed resins had inferior flexural and impact strength compared to heat-polymerized PMMA, which supports the notion that printed materials are currently less durable in critical mechanical tests [29,30]. Additionally, the different denture tooth types displayed varied fracture modes, which had a significant impact on their fracture resistance. This study observed two distinct types of damage: fractures that happened without visible deformation and those that occurred after deformation. Various material microstructures can lead to a range of responses to applied loads, spanning from what is essentially a “brittle mode” characterized by cracks to a more “quasi-plastic mode” marked by deformation dominance [31].

The chipping test revealed notable differences in fracture behavior among various denture tooth types. Notably, SpofaDent and Ivostar Shade teeth demonstrated impressive resilience, exhibiting strong fracture resistance under stress. However, their performance was accompanied by some deformation. Before reaching the critical fracture point, both SpofaDent and Major Super Lux teeth showed visible signs of strain, including the development of a distinct cone-shaped depression. This pre-fracture deformation suggests a degree of material plasticity, wherein the denture teeth undergo localized structural changes in response to external forces [32]. Similarly, another study found that materials with higher plasticity, like those used in SpofaDent and Major Super Lux, display considerable deformation under stress, contributing to their overall durability and resistance to chipping [33]. Such behavior underscores the complex interplay between material composition, mechanical properties, and the inherent ability of denture teeth to withstand mechanical stressors. Understanding these nuances is essential for optimizing denture design and material selection to enhance durability and longevity in clinical applications.

In the indirect tensile fracture resistance test, both 3D-printed resin and SpofaDent teeth demonstrated significant fracture strength, with SpofaDent specimens showing some quasi-plastic deformation before fracturing. However, 3D-printed resin, Major Super Lux, and Ivostar Shade teeth did not exhibit this quasi-plastic deformation. Notably, the fracture pattern for 3D-printed resin teeth differed from that of other denture types, displaying simultaneous fractures in both the buccal and lingual cusps, rather than the central line fracture observed in the other materials. This observation aligns with findings from De Angelis et al., who reported that certain conventional materials, including heat-cured acrylics, often exhibit similar quasi-plastic deformation properties, contributing to their robustness [18].

In recent studies, including that of Gad et al. [29], it has been established that 3D-printed denture bases offer significant advantages in precision and customization; however, they also exhibit varying mechanical strength and distinct fracture patterns, indicating potential limitations in durability. Similarly, our research highlights the differences in fracture resistance and overall performance of 3D-printed teeth, suggesting that, while these materials present innovative benefits, their mechanical behaviors may not align with those of conventional denture materials.

Typically made from photopolymer resins, 3D-printed resin denture teeth generally exhibit hardness levels that vary depending on the resin formulation and printing parameters. In this study, if the 3D-printed resin teeth displayed lower hardness compared to conventional materials, this might correlate with their increased susceptibility to chipping observed in the results. Lower hardness could suggest that while 3D-printed resins can achieve high precision and detailed anatomical features, they might not offer the same level of wear resistance as traditional materials. These findings align with other studies, such as that by Brenes et al., which reported that 3D-printed resins often have lower hardness compared to conventional materials, potentially affecting their long-term performance and resistance to mechanical stress [31].

By contrast, conventional heat-cured acrylic denture teeth are known for their higher hardness, which contributes to their durability and resistance to wear. The heat-curing process enhances the hardness and mechanical strength of these materials, making them more resistant to chipping and deformation. This observation is supported by findings from studies like that of De Angelis et al. (2024), which highlighted the superior hardness and wear resistance of conventional acrylics compared to some newer materials, including 3D-printed resins [18]. The superior hardness of conventional acrylic teeth often translates into better performance in terms of longevity and clinical efficacy.

There was also no evidence of a quasi-plastic depression pattern preceding fracture in the indirect tensile test. Nevertheless, the fracture strength of 3D-printed resin teeth was as high as that of the teeth with quasi-plastic deformation. This may be because traditional denture teeth are made from a combination of materials, such as enamel and dentin, while 3D-printed resin teeth are constructed entirely from a single material. This study found that denture teeth showing quasi-plastic deformation before fracturing had greater fracture resistance compared to those that did not display this pattern. This can be attributed to the deformed shape of the teeth, which enabled them to withstand the load for a longer duration. Previous research on conventional denture tooth materials during chipping fracture tests noted significant deformations in soft materials [33]. In that study, the material with the highest hardness had the lowest resistance to edge chipping and showed bulging deformation and initial splitting before the main piece chipped off, which is consistent with the findings of the present study [34]. Generally, rigid materials experienced a more sudden loss of strength compared to quasi-plastic materials [35,36]. The challenges surrounding chipping and fracturing in dental prosthetics persist despite the declining utilization of ceramic denture teeth [37]. These issues transcend traditional removable prostheses and extend into the domain of modern implant-supported solutions, highlighting the universal nature of the problem. The durability and resilience of denture prostheses are intricately linked to the chemical composition of their components, particularly the denture teeth and base materials [38].

3D printing technology provides notable benefits in denture production, especially regarding customization, speed, and accuracy. It enables the creation of highly individualized designs, allowing dentures to be specifically adapted to each patient’s unique anatomy for a more comfortable and precise fit [23]. Furthermore, the faster production process of 3D printing shortens the time needed for manufacturing compared to conventional methods, leading to quicker delivery of dentures. This reduction in wait time boosts patient satisfaction and improves overall treatment efficiency, offering a more streamlined and patient-focused approach to denture fabrication [29]. The financial benefits of 3D printing in denture manufacturing are considerable and diverse. Although the upfront cost of 3D printing equipment can be substantial, the technology delivers long-term savings by speeding up production, minimizing material waste, and reducing labor demands. Additionally, 3D printing enables higher precision and customization, which helps decrease the need for expensive adjustments and remakes, a common challenge with traditional manufacturing methods [5,11].

Despite its advantages, the widespread use of 3D printing in denture manufacturing faces challenges, including material limitations, inconsistent quality control, and high initial equipment costs, particularly for smaller practices [12]. If these issues are addressed, 3D printing could transform clinical practice by providing more durable, customized, and cost-effective dentures, reducing the need for frequent adjustments and improving patient comfort. However, ongoing research and standardization are necessary to integrate 3D printing into routine clinical use [31].

The biocompatibility of 3D-printed materials is typically tested through a series of standardized procedures, such as cytotoxicity tests, irritation studies, and sensitization tests, which assess the potential for these materials to cause harmful effects when in contact with oral tissues [6]. These tests are crucial for evaluating how 3D-printed resins interact with cells, tissues, and the immune system, ensuring their safety and suitability for long-term use in dental applications. However, the results of such tests for 3D-printed materials are still being explored, and more long-term studies are needed to fully understand their performance and safety in vivo [19].

The comparison of biocompatibility between 3D-printed materials and traditional ceramics reveals important differences and implications for clinical use. Traditional ceramic materials have long been favored for their excellent biocompatibility, durability, and aesthetic qualities, with minimal risk of irritation or allergic reactions and resistance to wear and staining. By contrast, 3D-printed resins, though promising, are still relatively new, and their biocompatibility can vary depending on the specific material used. Some 3D-printed resins meet biocompatibility standards like ISO 10993, but concerns about long-term degradation, chemical leaching, or tissue irritation remain [20,21]. Additionally, 3D-printed materials may not yet match the strength and wear resistance of ceramics, affecting their longevity under masticatory forces. While early data shows that 3D-printed materials could offer customization, cost, and production speed advantages, further research is needed to assess their long-term biocompatibility and performance in clinical settings. Until then, ceramics will remain the gold standard; but the potential for 3D-printed materials to offer comparable or even superior biocompatibility could lead to significant advancements in dental care [15].

The clinical implications regarding fracture resistance are notable for the long-term performance of removable dentures [36]. Our analysis indicates that the NextDent material demonstrates significantly higher mean fracture resistance values compared to Ivostar Shade and Major Super Lux, suggesting that NextDent dentures may offer superior durability and longevity in clinical use [39]. This enhanced fracture resistance could translate to fewer instances of denture breakage and longer intervals between replacements or repairs, ultimately improving patient satisfaction and reducing long-term costs for both patients and practitioners [40,41].

In addition, by developing denture teeth with improved fracture resistance and optimizing the interface between the teeth and the denture base, clinicians can better meet the evolving needs and expectations of patients [42]. Additionally, interdisciplinary collaboration between dentists, prosthodontists, materials scientists, and engineers is vital for driving innovation and fostering progress in this critical area of dental care. Ultimately, by leveraging scientific insights and technological innovations, the dental community can strive to provide patients with durable, functional, and aesthetically pleasing prosthetic solutions [43].

Denture teeth typically comprise polymethylmethacrylate (PMMA) or urethane dimethacrylate (UDMA) resins, each exhibiting distinct variations in minor components, filler sizes, and quantities [42]. These nuanced differences in composition profoundly influence the mechanical properties and fracture resistance of denture teeth, thereby impacting the overall durability and longevity of prosthetic devices. As such, understanding the intricacies of these materials and their formulations is paramount for optimizing the performance and reliability of denture prostheses in clinical practice [41,44]. Efforts to enhance material design and manufacturing processes hold promise for addressing these challenges and advancing the field of dental prosthodontics toward more resilient and long-lasting solutions [45,46].

Limited studies have focused on the biomechanical aspects of 3D-printed resin teeth [47]. The NextDent 3D-printing denture teeth resin is a specially developed material for additive manufacturing. As 3D printing technology continues to advance, and multi-layer artificial teeth become additively manufacturable in the future, a more comprehensive study would be necessary to assess fracture energy. Previous studies have compared the mechanical properties of various types of conventional denture teeth [46]. However, these tests have not fully characterized denture tooth fractures. The chipping fracture and indirect tensile tests employed in this study were previously used to examine the mechanical strengths of denture teeth [48].

The limitations of the current study include:

- Limited sample variation: This study focused on only four specific types of denture teeth. The limited variety of denture types examined in this study may not fully represent the wide range of materials and manufacturing techniques used in modern dental practice, potentially making the results less applicable to the broader market. This constraint could impact on the reliability of conclusions about the overall fracture resistance and performance of 3D-printed denture teeth. Furthermore, each denture type may have distinct material properties, meaning the findings might not be applicable to all types of dentures. Although expanding the sample was hindered by logistical factors, such as time, resources, and sample availability, future research should incorporate a wider variety of denture materials and production methods. A more diverse sample would provide a more thorough analysis and improve the generalizability of the results, offering deeper insights into how different materials and techniques affect denture performance.
- Lack of long-term durability assessment: The assessment of fracture resistance was conducted over a short duration, which does not account for long-term durability or the cumulative effects of repeated stress and wear. Understanding the longevity of 3D-printed denture teeth requires extensive longitudinal studies, which can be resource-intensive and time-consuming. Due to the inherent complexities of human factors, such as variations in patient behavior and oral environments, it becomes challenging to simulate a true long-term assessment within the confines of a single study. Future research should focus on conducting extensive longitudinal studies to assess the longevity and wear resistance of 3D-printed denture teeth over extended periods. These studies would provide a more accurate representation of the material’s performance in everyday use and help identify any potential issues that may arise over time. Furthermore, long-term studies would allow for better predictions of when denture replacements or adjustments may be needed, offering more reliable information for both clinicians and patients.
- Simplified testing conditions: The tests were performed under controlled laboratory conditions, which may not adequately replicate the multifaceted forces that denture teeth encounter during actual mastication. While it is feasible to create a more dynamic testing environment, such as simulating chewing forces, it introduces additional variables that could complicate the results. Creating a fully realistic testing scenario often requires sophisticated equipment and methodologies that may exceed budgetary and temporal constraints, hindering the ability to produce results that mirror real-world conditions accurately. Furthermore, fatigue loading tests, which are commonly used to assess the durability of dental materials under repetitive stress, were not included in this study. Fatigue loading is essential for understanding how denture teeth withstand long-term use, as materials can weaken and fracture over time due to repeated mechanical loading. Conducting such tests requires sophisticated equipment and methodologies, which may exceed budgetary and temporal constraints, making it challenging to produce results that accurately reflect real-world wear and tear. Therefore, while controlled laboratory testing provides valuable insights, it may not fully capture the complexities of actual patient use, and future research should incorporate fatigue loading tests to better simulate the long-term performance of 3D-printed denture materials.
- Artificial testing conditions compared to in vivo scenarios: While laboratory tests provide a controlled environment to evaluate fracture resistance, they fail to encompass the diverse factors influencing denture performance in real life. Oral hygiene practices, individual masticatory forces, and patient-specific anatomical variations play critical roles in the actual functioning of denture teeth. Conducting research in real-world scenarios presents logistical challenges and ethical considerations that may limit the feasibility of such studies. Moreover, the vast array of individual behaviors, dietary habits, and wear patterns complicates the ability to generalize findings. Hence, while this study lays a foundation for understanding 3D-printed denture teeth, further clinical investigations are necessary to validate these findings and assess material performance under real-life conditions. This knowledge is essential for guiding clinicians in selecting the most appropriate denture materials and ultimately improving patient outcomes. Potential bias in sample selection: There is a possibility of bias in the selection of denture teeth for each group, as certain brands or types may have been chosen based on availability or researcher preference. Randomized selection or blinding methods could help mitigate bias in future studies.
- Limited generalizability: The results might not apply universally to all categories of 3D-printed denture teeth or resin materials. Variations in factors like printing parameters, resin compositions, and post-processing methods could impact fracture resistance. Therefore, additional research encompassing a wider array of materials and manufacturing approaches is necessary for broader applicability.

Future research should address critical gaps in understanding the biomechanical properties of 3D-printed resin teeth. While promising materials like NextDent denture teeth resin have shown potential, their long-term performance under real-world conditions, including cyclic loading, thermal cycling, and chemical exposure, remains underexplored. As multi-layered artificial teeth become feasible through advanced 3D printing, studies should optimize material compositions and structures to enhance durability and fracture resistance. Comprehensive characterization, including tests for flexural strength, wear resistance, and fracture toughness, is necessary. Emerging techniques like digital image correlation (DIC) and finite element analysis (FEA) could provide deeper insights into stress distribution and failure mechanisms.

## 5. Conclusions

The incorporation of three-dimensional (3D) printed resin denture teeth represents a significant advancement in digital dentistry. This study evaluated the resilience of 3D-printed denture teeth to chipping and indirect tensile fractures, finding that 3D-printed teeth exhibited lower resistance to indirect tensile fractures than conventionally manufactured resin teeth. However, they demonstrated notable resilience against buccal chipping and distinct fracture patterns, suggesting structural integrity that merits further exploration. The findings point to a potential relationship between 3D printing parameters, material properties, and fracture behaviors, emphasizing the need for optimization to enhance performance. Future research should focus on improving resin formulations, refining printing parameters, and conducting long-term studies to assess durability under real-world conditions. These efforts could unlock the full potential of 3D-printed denture teeth, making them a durable, customizable, and cost-effective solution in modern prosthodontics.

## Figures and Tables

**Table 1:** Types of denture teeth used in this study.

Product	Composition	Detail	Manufacturer
NextDent	Methacrylate-based photopolymerized resin	3D printing	3D Systems, Soesterberg, The Netherlands
Ivostar Shade	MPM–PMMA *	Mold XS	Ivoclar Vivadent, Schaan, Liechtenstein
Spofadent™Plus	MPM–PMMA *	Mold XS	SpofaDental, Jičín, Czechia
Major Super Lux	MPM–PMMA *	Mold XS	Major Prodotti Dentari SPA, Moncalieri, TO, Italy

* MPM—multiplex polymer matrix; PMMA—polymethylmethacrylate.

**Table 2:** Tukey’s honestly significant difference post hoc analysis of fracture toughness of four types of artificial teeth.

(I) V2	(J) V2	Mean Values (I-J)	Standard Deviation	*p*	95%-Interval of Confidentiality	
					Lower	Upper
NextDent	Ivostar Shade	−0.346413 *	0.022049	<0.01	−0.28645	−0.17284
	Major Super Lux	−0.325271 *	0.022074	<0.01	−0.34831	−0.23442
	SpofaDent Plus	−0.478271 *	0.022087	<0.01	−0.40421	−0.29212
SpofaDent Plus	NextDent	0.429414 *	0.022089	<0.01	0.17563	0.28646
	Major Super Lux	−0.261857 *	0.022053	0.028	−0.11876	−0.00484
	SpofaDent Plus	−249657 *	0.022011	<0.01	−0.14758	−0.06286
Major Super Lux	NextDent	0.274571 *	0.022049	<0.01	0.23852	0.34834
	Ivostar Shade	0.141853 *	0.022061	0.028	0.00487	0.11887
	SpofaDent Plus	−0.158002 *	0.022061	0.045	−0.11508	−0.00094
Ivostar Shade	NextDent	0.429277 *	0.022063	<0.01	0.29226	0.40633
	Major Super Lux	0.314857 *	0.022063	<0.01	0.06286	0.17687
	Ivostar Shade	0.258300 *	0.022069	0.045	0.00096	0.11508

* The difference in means is significant at the 0.05 significance level.

## Data Availability

The data presented in this study are available within the article.

## References

[1] Gad M.M., Al-Thobity A.M., Rahoma A., Abualsaud R., Al-Harbi F.A., Akhtar S. (2019). Reinforcement of PMMA Denture Base Material with a Mixture of ZrO_2_ Nanoparticles and Glass Fibers. Int. J. Dent..

[2] Cevik P., Yildirim-Bicer A.Z. (2018). The effect of silica and prepolymer nanoparticles on the mechanical properties of denture base acrylic resin. J. Prosthodont..

[3] Sun J., Forster A.M., Johnson P.M., Eidelman N., Quinn G., Schumacher G., Zhang X., Wu W.-L. (2011). Improving performance of dental resins by adding titanium dioxide nanoparticles. Dent. Mater..

[4] Nejatian T., Nathwani N., Taylor L., Sefat F. (2020). Denture Base Composites: Effect of Surface Modified Nano- and Micro-Particulates on Mechanical Properties of Polymethyl Methacrylate. Materials.

[5] Altarazi A., Haider J., Alhotan A., Silikas N., Devlin H. (2022). Assessing the Physical and Mechanical Properties of 3D Printed Acrylic Material for Denture Base Application. Dent. Mater..

[6] Karacaer O., Polat T.N., Tezvergil A., Lassila L.V., Vallittu P.K. (2003). The effect of length and concentration of glass fibers on the mechanical properties of an injection- and a compression-molded denture base polymer. J. Prosthet. Dent..

[7] Ellakwa A.E., Morsy M.A., El-Sheikh A.M. (2008). Effect of Aluminum Oxide Addition on the Flexural Strength and Thermal Diffusivity of Heat-Polymerized Acrylic Resin. J. Prosthodont..

[8] Kattadiyil M.T., Goodacre C.J., Baba N.Z. (2012). CAD/CAM Complete Denture Systems: An Overview of Clinical Relevance. J. Prosthodont..

[9] Dimitrova M., Vlahova A., Kalachev Y., Zlatev S., Kazakova R., Capodiferro S. (2023). Recent Advances in 3D Printing of Polymers for Application in Prosthodontics. Polymers.

[10] Wang R., Tao J., Yu B., Dai L. (2014). Characterization of multiwalled carbon nanotube-polymethyl methacrylate composite resins as denture base materials. J. Prosthet. Dent..

[11] Bacali C., Badea M., Moldovan M., Sarosi C., Nastase V., Baldea I., Chiorean R.S., Constantiniuc M. (2019). The influence of graphene in the improvement of physico-mechanical properties in PMMA Denture Base Resins. Materials.

[12] Zafar M.S. (2020). Prosthodontic Applications of Polymethyl Methacrylate (PMMA): An Update. Polymers.

[13] (2020). Standard Test Method for Tensile Properties of Plastics.

[14] (2018). Dentistry—Polymer-Based Restorative Materials.

[15] Gad M.M., Fouda S.M., Abualsaud R., Alshahrani F.A., Al-Thobity A.M., Khan S.Q., Akhtar S., Ateeq I.S., Helal M.A., Al-Harbi F.A. (2022). Strength and Surface Properties of a 3D-Printed Denture Base Polymer. J. Prosthodont..

[16] Said M.M., Elhassan M., Sadek M., Ahmed H.B., Abd El-Ghany M., Mounir A., Ibrahim S. (2020). Mechanical Properties of 3D-Printed vs. Conventional Denture Base Materials: A Systematic Review. J. Prosthet. Dent..

[17] Khan S.Q., Alharbi F.A., Alqurashi M., Alzahrani A., Althobity A.M., Akhtar S., Ateeq I.S., Gad M.M. (2021). Comparative Evaluation of Mechanical Properties of 3D Printed and Conventional PMMA Denture Bases: An In Vitro Study. J. Prosthodont..

[18] De Angelis F., D’Amario M., Jahjah A., Frascaria M., Vadini M., Sorrentino E., Biferi V., D’Arcangelo C. (2024). Flexural Properties of Three Novel 3D-Printed Dental Resins Compared to Other Resin-Based Restorative Materials. Prosthesis.

[19] Mann R.S., Ruse N.D. (2022). Fracture toughness of conventional, milled and 3D printed denture bases. Dent. Mater..

[20] (2018). Biological Evaluation of Medical Devices—Part 1: Evaluation and Testing Within a Risk Management Process.

[21] Gao J., Patterson B.A., Kashcooli Y., O’Brien D., Palmese G.R. (2022). Synergistic fracture toughness enhancement of epoxy-amine matrices via combination of network topology modification and silica nanoparticle reinforcement. Compos. Part B Eng..

[22] Srinivasan M., Kalberer N., Kamnoedboon P., Mekki M., Durual S., Özcan M., Müller F. (2021). CAD-CAM Complete Denture Resins:An Evaluation of Biocompatibility, Mechanical Properties, and Surface Characteristics. J. Dent..

[23] Gad M.M., Alalawi H., Akhtar S., Al-Ghamdi R., Alghamdi R., Al-Jefri A., Al-Qarni F.D. (2023). Strength and Wear Behavior of Three-Dimensional Printed and Prefabricated Denture Teeth: An In Vitro Comparative Analysis. Eur. J. Dent..

[24] (2017). Standard Test Method for Splitting Tensile Strength of Cylindrical Concrete Specimens.

[25] Saponaro P.C., Yilmaz B., Heshmati R.H., Mcglumphy E.A. (2016). Clinical performance of CAD-CAM-fabricated complete dentures: A cross-sectional study. J. Prosthet. Dent..

[26] Lee H.H., Lee C.J., Asaoka K. (2012). Correlation in the Mechanical Properties of Acrylic Denture Base Resins. Dent. Mater. J..

[27] Helal M.A., Albayatti N., Elwazir M.Y., Elwan M. (2022). Comparative Evaluation of Surface Roughness, Impact Strength, and Hardness of Milled and 3D-Printed Denture Base Materials. Polymers.

[28] Alshabib A., Silikas N., Watts D.C. (2019). Hardness and fracture toughness of resin-composite materials with and without fibers. Dent. Mater..

[29] Al-Harbi F.A., Abdel-Halim M.S., Gad M.M., Fouda S.M., Baba N.Z., AlRumaih H.S., Akhtar S. (2018). Effect of nanodiamond addition on flexural strength, impact strength, and surface roughness of PMMA denture base. J. Prosthodont..

[30] Nejatian T., Johnson A., van Noort R. (2006). Reinforcement of denture base resin. Adv. Sci. Technol..

[31] Brenes C., Bencharit S., Fox T. (2023). Evaluation of Prosthetic Outcomes and Patient Satisfaction with 3D-Printed Implant-Supported Fixed Prosthesis. Cureus.

[32] Balos S., Pilic B., Markovic D., Pavlicevic J., Luzanin O. (2014). Poly (methyl-methacrylate) nanocomposites with low silica addition. J. Prosthet. Dent..

[33] Nobrega A.S., Andreotti A.M., Moreno A., Sinhoreti M.A., dos Santos D.M., Goiato M.C. (2016). Influence of adding nanoparticles on the hardness, tear strength, and permanent deformation of facial silicone subjected to accelerated aging. J. Prosthet. Dent..

[34] Vallittu P.K., Vojtkova H., Lassila V.P. (1995). Impact strength of denture polymethyl methacrylate reinforced with continuous glass fibers or metal wire. Acta Odontol. Scand..

[35] Nakamura M., Takahashi H., Hayakawa I. (2007). Reinforcement of denture base resin with short-rod glass fiber. Dent. Mater. J..

[36] Cleto M.P., Silva M.D.D., Nunes T.S.B.S., Viotto H.E.C., Coelho S.R.G., Pero A.C. (2022). Evaluation of shear bond strength between denture teeth and 3D-printed denture base resin. J. Prosthodont..

[37] Alhallak K., Hagi-Pavli E., Nankali A. (2023). A Review on Clinical Use of CAD/CAM and 3D Printed Dentures. Br. Dent. J..

[38] Cha H.S., Park J.M., Kim T.H., Lee J.H. (2020). Wear resistance of 3D-printed denture tooth resin opposing zirconia and metal antagonists. J. Prosthet. Dent..

[39] Alharbi N., Osman R., Wismeijer D. (2016). Factors Influencing the Dimensional Accuracy of 3D-Printed Full-Coverage Dental Restorations Using Stereolithography Technology. Int. J. Prosthodont..

[40] Javaid M., Haleem A. (2019). Current Status and Applications of Additive Manufacturing in Dentistry: A Literature-Based Review. J. Oral Biol. Craniofac. Res..

[41] Nakamura K., Harada A., Inagaki R., Kanno T., Niwano Y. (2019). Milled vs. Printed Dentures: A Comparative Analysis of Fracture Toughness and Impact Strength. J. Dent. Res..

[42] Tahayeri A., Morgan M.C., Fugolin A.P.P., Bompolaki D., Athirasala A., Almutairi M., Ferracane J.L., Bertassoni L.E. (2018). 3D Printed versus Conventionally Cured Provisional Crown and Bridge Dental Materials. Dent. Mater..

[43] Bilgin M.S., Baytaroğlu E.N., Erdem A., Dilber E. (2016). A Review of Computer-Aided Design/Computer-Aided Manufacture Techniques for Removable Denture Fabrication. Eur. J. Dent..

[44] Goodacre B.J., Goodacre C.J., Baba N.Z., Kattadiyil M.T. (2016). Comparison of Denture Base Adaptation between CAD-CAM and Conventional Fabrication Techniques. J. Prosthet. Dent..

[45] Zandinejad A., Floriani F., Lin W.S., Naimi-Akbar A. (2024). Clinical Outcomes of Milled, 3D-Printed, and Conventional Complete Dentures in Edentulous Patients: A Systematic Review and Meta-Analysis. J. Prosthodont..

[46] Steinmassl P.A., Wiedemair V., Huck C., Klaunzer F., Steinmassl O., Grunert I., Dumfahrt H. (2018). Do CAD/CAM Dentures Really Shift the Paradigm? A Clinical Crossover Trial. J. Dent..

[47] Kalberer N., Mehl A., Schimmel M., Müller F. (2019). CAD-CAM Removable Complete Denture Fabrication for Edentulous Patients: A Case Report of a Novel Clinical Protocol. Int. J. Prosthodont..

[48] Revilla-León M., Özcan M. (2019). Additive Manufacturing Technologies Used for Processing Polymers: Current Status and Potential Application in Prosthetic Dentistry. J. Prosthodont..

